# Exploring the Role of Non-synonymous and Deleterious Variants Identified in Colorectal Cancer: A Multi-dimensional Computational Scrutiny of Exomes

**DOI:** 10.2174/0113892029285310231227105503

**Published:** 2024-01-22

**Authors:** Chandrashekar Karunakaran, Vidya Niranjan, Anagha S. Setlur, Dhanya Pradeep, Jitendra Kumar

**Affiliations:** 1 Department of Biotechnology, R V College of Engineering, Bangalore, 560059, affiliated to Visveswaraya Technological University, Belagavi, 590018, India;; 2 Department of Biotechnology, BMS College of Engineering, Bangalore, 560019, India;; 3 Biotechnology Industry Research Assistance Council (BIRAC), CGO complex Lodhi Road, New Delhi, India

**Keywords:** Colorectal cancer, exome analysis, non-synonymous, deleterious mutations, mutational profiling, multi-dimensional genomics, CTSB, CPNE1

## Abstract

**Introduction:**

Colorectal cancers are the world’s third most commonly diagnosed type of cancer. Currently, there are several diagnostic and treatment options to combat it. However, a delay in detection of the disease is life-threatening. Additionally, a thorough analysis of the exomes of cancers reveals potential variation data that can be used for early disease prognosis.

**Methods:**

By utilizing a comprehensive computational investigation, the present study aimed to reveal mutations that could potentially predispose to colorectal cancer. Ten colorectal cancer exomes were retrieved. Quality control assessments were performed using FastQC and MultiQC, gapped alignment to the human reference genome (hg19) using Bowtie2 and calling the germline variants using Haplotype caller in the GATK pipeline. The variants were filtered and annotated using SIFT and PolyPhen2 successfully categorized the mutations into synonymous, non-synonymous, start loss and stop gain mutations as well as marked them as possibly damaging, probably damaging and benign. This mutational profile helped in shortlisting frequently occurring mutations and associated genes, for which the downstream multi-dimensional expression analyses were carried out.

**Results:**

Our work involved prioritizing the non-synonymous, deleterious SNPs since these polymorphisms bring about a functional alteration to the phenotype. The top variations associated with their genes with the highest frequency of occurrence included *LGALS8*, *CTSB*, *RAD17*, *CPNE1*, OPRM1, *SEMA4D*, *MUC4*, *PDE4DIP*, *ELN* and *ADRA1*A. An in-depth multi-dimensional downstream analysis of all these genes in terms of gene expression profiling and analysis and differential gene expression with regard to various cancer types revealed *CTSB* and *CPNE1* as highly expressed and overregulated genes in colorectal cancer.

**Conclusion:**

Our work provides insights into the various alterations that might possibly lead to colorectal cancer and suggests the possibility of utilizing the most important genes identified for wet-lab experimentation.

## 1 INTRODUCTION

With increasing incidences of cancer globally, colorectal cancer currently stands as the third most commonly diagnosed type of cancer. As of 2022, the American Cancer Society evaluations for the number of colorectal cancers in the United States were 1,06,180 fresh cases of colon cancer and 44,850 new cases of rectal cancer [1], with deaths estimated to be about 52,580 including both men and women. Despite there being several screening techniques for the prevention of colorectal cancer and treatment strategies such as surgery, chemotherapy and radiation for colorectal cancer, it still lacks a suitable prognosis. Exomes are the coding region of a gene. A thorough analysis of the exomes of cancers reveals potential variation data that can be used for early disease prognosis. Mutations in genes such as driver genes, tumor suppressor genes, proto-once and oncogenes trigger cancers by causing DNA damage and genetic instability [2].

Currently, pursuing recurring variations depending on the frequency by which a gene is altered is the current strategy for the analysis of cancer exome sequences. Some other techniques include analyzing the predicted score for the impact the mutations have on the structure or function of a protein and identifying the spatial clusters of variations with respect to each residue [3]. Moreover, the identification of all cancer-causing genes from the exomes of cancers is a huge task suffused with numerous challenges. There still remain uncertainties as to the constitution of specific cancer-causing genes. Therefore, the lacunae existing currently in cancer exome research can be filled by a thorough investigation of all genes in the exome, including different mutations such as somatic and germline alterations. Thus, high-throughput analyses of cancer exomes using* in-silico *strategies are now paving the way for swift mutation identification and analysis.

The demerits of Sanger sequencing [4] allowed for the development of next-generation sequencing technology (NGS) that sequences several thousand samples simultaneously at high accuracy and precision [5]. Advancements in this technology have now paved the way for the identification of rare somatic and germline mutations [6]. With whole exome sequencing (WES) being utilized for the detection of cancers, the identification of prognostic and diagnostic biomarkers also becomes an essential part of this process. It is crucial in the design of suitable treatment strategies as well as early recognition of the disease. Since the detection of cancer and its prognosis is dependent on the understanding and analysis of the molecular pathways involved, there is a great need to unravel this data through the examination of existing cancer exomes to primarily broaden the prospect of early detection and early treatment.

At present, for colorectal cancer, genes such as *KRAS, BRAF* and *APC* are considered to be reliable markers that point towards its detection [7]. Additionally, *MSI* (microsatellite instability) is a prognostic marker for colorectal cancer that corresponds to a phenotype observed in colorectal cancer when genes of the mismatch repair pathway are altered [8]. This shows that the identification of mutations is essential for the detection of any potential markers. Moreover, non-synonymous germline variations that bring about a significant alteration to the function of a protein can be pivotal in determining genes that could predispose to colorectal cancer. These alterations could also serve as potential susceptibility markers, particularly while screening for a panel of variants that might incrementally contribute to the increased risk of colorectal cancer, suggesting the cumulative burden of these variants. Determination of SNPs that regulate gene expression in driver genes is crucial to understanding the complex mechanisms underlying colorectal cancer, thereby enabling early diagnosis and improved treatment outcomes. Therefore, the present study focused on extensively investigating 10 colorectal cancer exomes for the identification of different variations that could potentially point towards the disease and aid in early detection.

## 2 MATERIALS AND METHODS

### 2.1 Dataset Retrieval

Colorectal cancer exome sample exomes for ten NGS sequenced samples were retrieved from the publicly available NCBI SRA (National Centre for Biotechnology Information, Sequence Read Archive) database [9]. For comparative analysis, the human reference genome hg19 (Genome Reference Consortium Human Reference 19) was also retrieved using the Genome Reference Consortium database (https://www.ncbi.nlm.nih.gov/grc/human). All the sequence data files were downloaded using sra-toolkit in.SRA format and then converted to fastq. These were then split into forward and reverse reads. Table **1** details the various colorectal cancer exomes that were used in the study.

### 2.2 Pre-processing of Raw Data

All ten colorectal cancer exomes were pre-processed prior to the calling of variants.

#### 2.2.1 Quality Control Checks for Raw Data

FastQC (https://github.com/sadrews/FastQC) and MutiQC (https://github.com/ewels/MultiQC) [8, 9] offer a straightforward yet effective approach to conducting quality assessments on raw sequence data. In a comprehensive review of tools for examining and verifying the quality of cancer genome sequencing data previously, it was identified that the preferred tools for evaluating the quality of these genomes, including exome sequencing data for comprehensive somatic variant calling in human cancer genomes, are FastQC and MultiQC [10]. Consequently, an analysis of mean sequence quality per read and per base, nucleotide content per base position, GC distribution, *etc*., was carried out using FastQC on the raw data sequences in fastq format. Therefore, the mean sequence quality per reading and per base, GC content distribution, nucleotide content per position of the base, adaptor content, *etc*., were all checked using FastQC with input as the raw sequences in fastq format. The quality checks output obtained as HTML reports were scrutinized and analyzed. MultiQC was then employed to obtain cumulative results for quality checks for all ten sequences, which also allowed for better visualization. The HTML reports from FastQC were uploaded, and MultiQc was run parallelly for all 10 raw data sequences. The final output generated showed the cumulative summary output for all the sequences, and the quality for each selected sequence was analyzed in terms of three categories: pass, warning and fail, using the log files obtained from FastQC quality runs for each of the forward and reverse reads.

#### 2.2.2 Gapped Alignment and File Conversion

Due to its sensitivity, greater output accuracy and higher speed of operation, Bowtie 2 (https://github.com/BenLangmead/bowtie2) was utilized for the gapped alignment of the sequences [11]. This tool employs the Burrow-Wheeler Transformation (BWT) algorithm, which was used for gapped alignment with the human reference genome hg19. Bowtie 2 uses a combination of BWT and the Smith-Waterman algorithm for performing gapped alignment. Thus, gapped alignment was performed for all ten cancer exome sequences to map the pre-processed reads to the reference. The bowtie2 index files for the genome were first built followed by which the forward and reverse reads were aligned to the reference genome. The output in SAM format was then converted to BAM format using SAMtools (https://github.com/samtools). SAMtools stands out as a widely employed tool for processing data derived from high-throughput sequencing. With enhanced speed and an improved capacity to index files, it facilitates the swift sorting and creation of BAM files [12]. Consequently, in the current investigation, the reads underwent sorting, recalibration of quality scores, realignment of indels, and filtering of the reads using SAMtools.

The alignment qualities were further improved to reduce the occurrence of false variant calls by taking the aligned sequences through several refinement steps. In the current study, conversion of the file from SAM to BAM, sorting of the BAM files and merging were performed for all ten aligned sequences. BAM files act as the binary, compressed files to SAM that are easier to retrieve and use [13]. This conversion was performed since BAM has a compact size and allows for quicker retrieval of the aligned sequences. The outputs obtained were analyzed prior to variant calling.

### 2.3 Processing and Variant Calling

Processing and calling of variants were carried out to identify the mutations from the sequenced data. Processing of the variants was performed using PICARD, and the variants were called using Haplotype caller from the gold standard method of the GATK pipeline (https://github.com/broadinstitute/gatk, The Genome Analysis Toolkit) [14, 15]. The GATK pipeline, established as the gold standard method since its initial publication in 2010, is renowned for its reliability, with an impressive F-score of 0.978, representing the harmonic mean of precision and recall [16]. Moreover, GATK is recognized for its ability to identify potential variants across diverse sequencing platforms and experimental designs [17], excelling particularly in the accurate discovery of true SNPs in exome datasets. The PCR duplicates were marked using PICARD, and GATK was employed for recalibration of the base quality and local realignment with BAM files as the input. Analysis of the co-variates was carried out by building the BAM indexes, SortSam and base recalibration. Once this was completed, the mutants were called. Outputs were obtained as VCF (Variant calling files) files, and these were scrutinized.

The SNPs that were detected were then filtered and annotated using the snpEFF (http://pcingola.github.io/SnpEff/) open-source tool that is platform-independent, flexible and multi-organism compatible [18]. The output VCF files were obtained after running snpEFF for all ten exomes, and the HTML summary files were then analyzed.

### 2.4 Post-processing of Variants and Mutational Profiling

SIFT (https://sift.bii.a-star.edu.sg/) was utilised to further process the annotated variants [19]. Results from SIFT tool were also cross-verified using PolyPhen2 (https://github.com/hammerlab/vcf-annotate-polyphen), which also works on the same principle, but classifies the mutations as “benign,” “possibly damaging,” and “probably damaging” [20]. The SIFT protocol assesses whether the amino acid substitution responsible for a variant in cancer exome data affects the protein's function. Utilizing sequence homology, the SIFT algorithm performed predictions on the potential effects of all substitutions at each position in the protein sequence [21]. Batch query files containing information about the chromosome, position of the variant and the specific nucleotide alteration were prepared for each dataset and used as input files for PolyPhen2. Verification was conducted by cross-referencing the results using PolyPhen2 (https://github.com/hammerlab/vcf-annotate-polyphen) [20]. PolyPhen2 operates on a similar principle, classifying identified variants into categories such as “possibly damaging,” “probably damaging,” and “benign” [22]. The results obtained were examined thoroughly, and a mutational profile for the same was developed.

A study published in 2018 highlighted the sensitivity and specificity of tools such as SIFT, PolyPhen and MutationTaster2 [23]. This study identified SIFT to have the highest accuracy and sensitivity (1.0), while Polyphen2 was slightly on the lower sensitivity side. However, another study noted that PolyPhen-2 and SIFT both have a high median sensitivity of 0.90 and 0.85, respectively and have similar median specificity values [24]. Therefore, in the current study, since PolyPhen2 was used for cross-referencing and verification of SIFT results, both these tools were employed together to help produce more reliable and reproducible results.

The exome analysis pipeline was followed by our previous study, Padmavathi *et al.*, 2021 [25].

### 2.5 Downstream Expression Analysis of Important Mutations

A thorough analysis of all the processed mutations from each of the ten datasets was performed to uncover common and most frequently occurring alterations. The mutations that occurred >= 10 times in each dataset and the overlapping variants across all exomes were examined and screened. A frequency filter was used to screen these and identify the variants. Studies have previously shown that when Haplotype caller from GATK is used for calling variants, germline and somatic can be distinguished from each other by choosing the frequency of occurrence of the variants. A study used 80% or greater allele frequency for classifying as germline and taking it forward for further analysis [26]. In the present work, the authors chose to filter out the variants occurring in most of the exome samples selected since, in the future, using them to point towards a cancer risk would be much easier.

It was later found after mutational profiling that all these mutations belonged to two major categories: non-synonymous and deleterious, which were then shortlisted for downstream gene expression profile analysis using various tools to comprehend the possibility of these genes acting as indicators that predispose to colorectal cancer.

The differential gene expression and comparisons of the expression levels of each of these identified genes were performed to obtain better insights into the potential downstream activities that could reveal those genes with potential cancer risk probabilities that can be explored further from wet lab studies.

#### 2.5.1 Gene Expression Profiling Using GEPIA

A gene expression profiling tool called GEPIA (Gene Expression Profiling Interactive Analysis, http://gepia.cancer- pku.cn/index.html) [27] was utilized for the top identified genes that occurred most frequently throughout all ten colorectal cancer exomes. This tool provides functions that are customizable, such as normal/tumor differential expression analysis, patient survival scrutiny, profiling as per types of cancers, correlation analysis, *etc*. In the present work, the gene “symbol” was provided in the webserver, and a single gene analysis was carried out in the form of box plots. The |Log_2_FC| threshold value was maintained at 1, the jitter size at 0.4 and the *p*-value cut-off to 0.01. The expression profile was studied for all the potential genes with respect to colon adenocarcinoma and rectum adenocarcinoma present in the GEPIA database. Once the box plots were obtained, these were analyzed for all the selected genes. The overall survival plots were also generated with a median group cut-off and at a 95% confidence interval. All other parameters, such as hazard ratios and axis units, were set to default. Moreover, a comparative multiple gene expression analysis was also performed to compare the expression of all frequently occurring genes in all ten colorectal cancer exomes. Colon adenocarcinoma and rectum adenocarcinoma were selected as the exomes for this differential gene expression. A heat chart was obtained that revealed which of the genes were predominantly expressed in each cancer type. These results were analyzed.

#### 2.5.2 Differential Expression of Genes in Various Cancer Types

OncoMX (https://www.oncomx.org/) [28] was utilized To understand and compare the expression of each of these genes in colorectal cancer and in other cancer types,. This comparative study will help provide a potential possibility of these genes being overexpressed in other cancer types as well, thereby giving a wider possibility for the detection of a cancer marker. The gene symbols are keyed in as the input for each identified gene, for which upregulation and down-regulation plots are generated with regard to different cancer types. This differential expression was studied thoroughly, and the results obtained helped in deciphering important colorectal cancer indicators.

#### 2.5.3 Multi-dimensional Gene Analysis

To further obtain an understanding of the potential genes that incline towards causing colorectal cancer, the most frequent genes were then subjected to a multi-dimensional gene analysis. For this purpose, cBio Cancer Genomics Portal was utilized (http://cbioportal.org)_to interactively explore the various genetic alterations and link it to the clinical outcomes [29, 30]. Graphical representations, network analysis, and visualizations were analyzed. All identified genes that showed potential for pointing towards colorectal cancer were queried as the input against all available colorectal adenocarcinoma studies in the cBioPortal database. From the 13 exomes available on colorectal cancer in cBioPortal, all 4535 patient samples were analyzed and run against our queried genes. From the results obtained, cancer type summary for all genes together, plots of mRNA expression against mutations and the various types of mutations that these genes have caused in different colorectal cancer samples.

With all this data, the potential influencers of colorectal cancer that require further analysis may be identified. This entire protocol used in the study is illustrated in Fig. (**1**).

### 2.6 Multi-variate Analysis Using Linear Regression

To establish the correlation between the gene frequencies and the gene expression data identified in the present study, a multi-variate analysis was performed. Linear regression using the least squares method [31] was employed, with 4 estimated parameters. These were:

β0- intercept, β1- gene expression, β2- gene frequency, and β3- correlation between gene expression and frequency.

The analysis was carried out using PRISM 9.0 [32]. The results obtained were scrutinized.

## 3 RESULTS

### 3.1 Pre-processing of Raw Data

All 10 colorectal cancer exomes were downloaded successfully and pre-processed to obtain enhanced quality data.

#### 3.1.1 Quality Control Checks

**Fig. (2):**
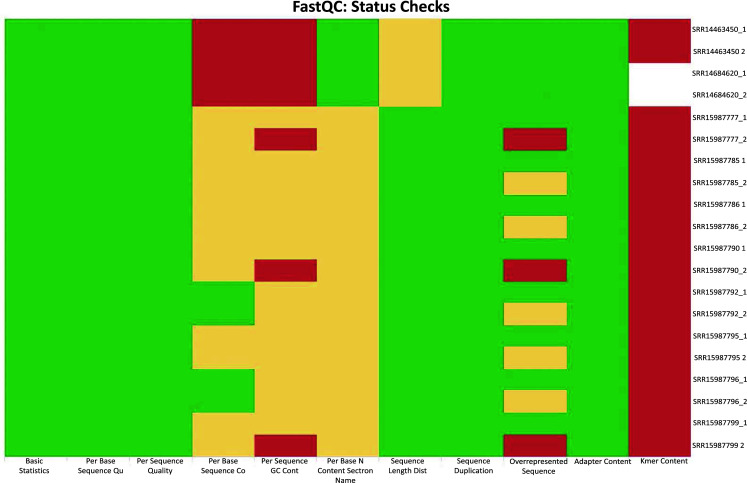
MultiQC status checks summarizing all FastQC results for 10 colorectal cancer exomes. Green represents very good quality calls, orange represents reasonable quality and red indicates poor quality and failure of the test. All 20 sequence files (duplicates of all 10 ten exomes) passed the per sequence quality scores check. The per sequence GC content of the 20 sequence files resulted in 13 files falling in the warning category while 7 failed the test. All the sequences formed a normal distribution with the peak of the curve at the mean GC content for *Homo sapiens*, with a deviation, causing FastQC to fail for the seven files at this step. Four samples passed the per base N content test, while a warning was raised for the remaining. Moreover, all the samples cleared the duplication levels, with most sequences falling into the far left of the plot.

FastQC checks for the ten colorectal cancer exomes showed that all the exomes used for this study were of good quality and could be used for further processing. None of the exomes required adapter trimming. The GC content of all exomes fell in the acceptable range between 40-60%, with SRR15987799 having a maximum GC content of 49%. In addition, all the exomes cleared the duplication levels and did not fall in the warning category. It was also found that the per sequence quality scores were all in the permissible range for all 10 exomes, and none of the exomes were marked with a warning for this parameter. The MultiQC summary report of all the ten exomes, including the individual forward and reverse read files, indicated that all the sequences had a Phred score greater than 20 with an error rate of 0.1% to 1% and an accuracy of ~99%. Further, all the 20 sequence files (duplicates of all 10 ten exomes) passed the per sequence quality scores check. The per sequence GC content of the 20 sequence files resulted in 13 files falling in the warning category while 7 failed the test. All the sequences formed a normal distribution with the peak of the curve at the mean GC content for *Homo sapiens*, with a deviation, causing FastQC to fail for the seven files at this step. Four samples passed the per base N content test, while a warning was raised for the remaining. It was further noticed that all the samples cleared the duplication levels, with most sequences falling into the far left of the plot. All 20 samples cleared the adapter content test, requiring no further trimming of the specific adapters (Fig. **2**).

Further, typically, to take forward the sequences for further analysis, basic statistics, per base sequence, per base quality, GC content and N content are important. To add to this, MultiQC runs duplicates of the sequences to make sure the results are accurately predicted. In our study, most of the sequences passed these quality checks. The red regions observed in the last column of Fig. (**2**) indicate the kmer content, which is generally not as important as the rest. Since an overall result showed that the sequences were of good quality, they were analyzed further. The raw MultiQC HTML files are provided as supplementary to corroborate our outcomes and provide more clarity on the same. These can be accessed in Supplementary file **S1**.

### 3.2 Variant Calling

**Fig. (3):**
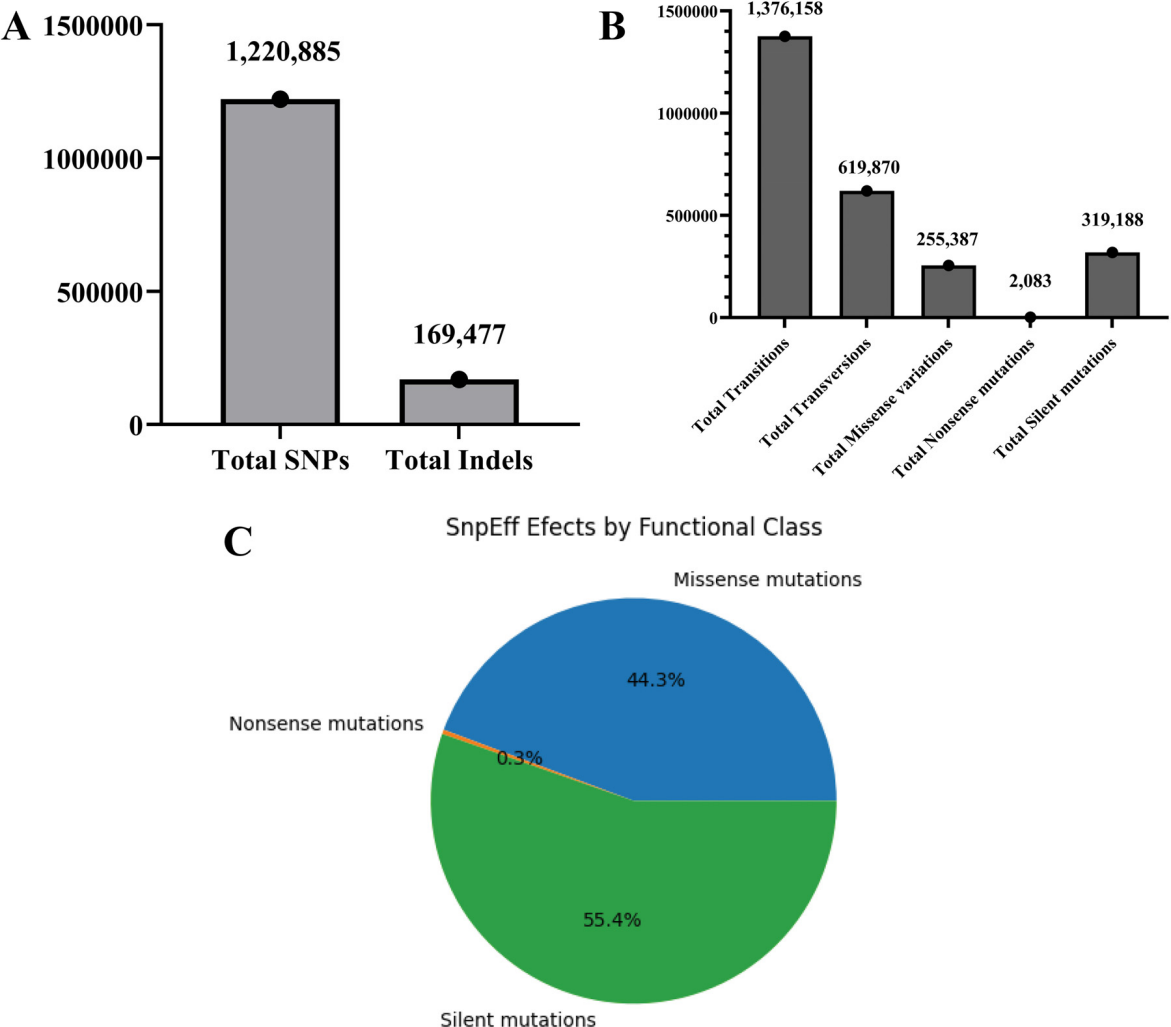
Variant calling data showing the GATK processing and annotation *via* snpEFF. **3A**) the total number of SNPs and indels that were obtained after variant calling *via* the GATK pipeline. Totally, 1,220,885 SNPs were detected, with 169,477 indels. **3B**) Annotated variants showing the total number of transitions (1,376,158), transversions (619,870), missense variations (255,387), nonsense mutations (2083) and silent mutations (319,188). **3C**) Based on the functional classes, 44.3% of the variants called were missense mutations, 55.4% were silent, and only 0.3% were found to be nonsense mutations.

The VCF files generated for each of the exomes using the GATK pipeline revealed the total number of SNPs and indels, as observed in the bar graph (Fig. **3A**). Each dataset was subjected to variant filtration, followed by which the final number of SNPs and indels were recorded. Dataset SRR15987799 contained the highest number of SNPs (188,833) and indels (27,331). Overall, from all 10 colorectal cancer exomes, 1,220,885 SNPs were identified, 169,477 indels, all totaling up to 1,390,362 SNPs. Additionally, information regarding the number of variants processed, variant rate details, number of effects by type, region and functional class (missense, nonsense and silent mutations), and the Ts/Tv (transitions/ transversions) ratio were also obtained, represented in the bar graph (Fig. **3B**). SnpEff annotation revealed a total of 1,376,158 transitions, 619,870 transversions, 255,387 missense variations, 2083 nonsense mutations and 319,188 silent mutations. From this, dataset SRR15987799 had a maximum number of transitions (221,059), and transversions (108,426). From both these results, it is understood that dataset SRR15987799 mutated more than the rest, warranting further analysis into it for scouting possible potential genes that may get overexpressed.

Based on the functional classes, 44.3% of the variants were missense mutations, 55.4% were silent, and only 0.3% were found to be nonsense (Fig. **3C**). With this variation data, finding the frequently occurring mutations with implications for colorectal cancer was much simpler. More details are provided in Supplementary file **S2**. SIFT annotations summary 1 and 2, PolyPhen2 annotations summary, SNPs and Indels called during GATK processing, SnpEff annotations summary.

### 3.3 Variant Post-processing and Mutational Profile Analysis

**Fig. (4):**
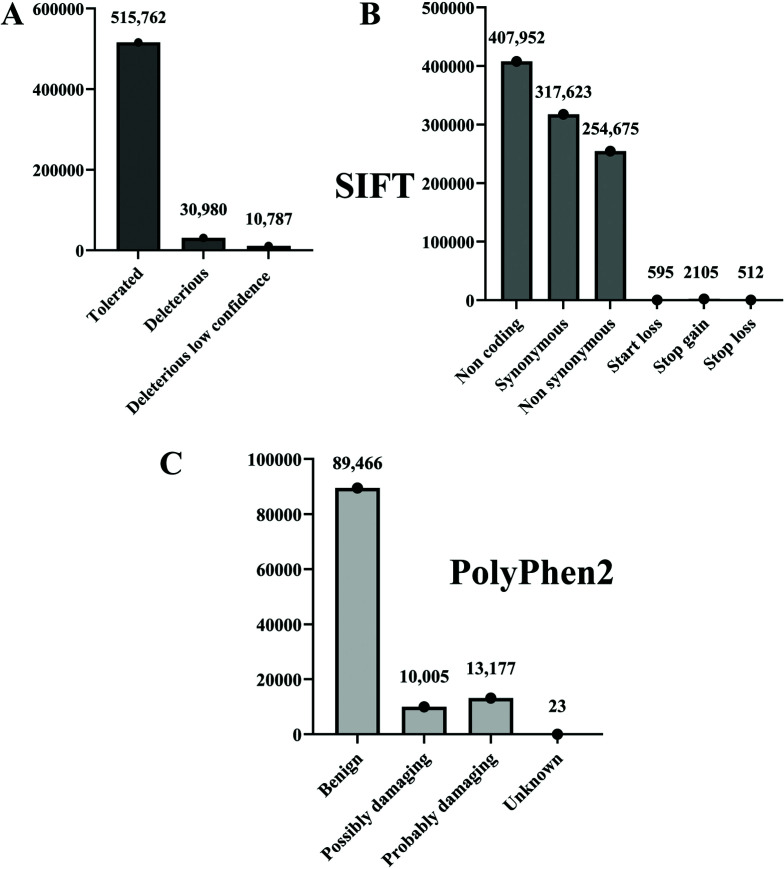
Variant post-processing and mutational profile analysis. **4A**) The SIFT annotation summary revealed a total of 515,762 tolerated variants, 30,980 deleterious variants and 10,787 deleterious low-confidence mutations. **4B**) 407,952 non-coding variants were observed, along with 317,623 synonymous, 254,675 non-synonymous, 595 starts lost, 2105 stop gain and 512 stop loss alterations. **4C**) A total of 89,466 variants were benign, 10,005 were possibly damaging, and 13,177 were found to be probably damaging, according to PolyPhen 2.

The SIFT annotation summary revealed a total of 515,762 tolerated variants, 30,980 deleterious variants and 10,787 deleterious low-confidence mutations (Fig. **4A**). The tool predicts the maximum number of mutations in the conserved regions and classifies it as deleterious or tolerated. Thus, dataset SRR15987790 had a maximum number of tolerated, deleterious and deleterious low-confidence mutations, different from the dataset that previously showed a higher number of SNPs and indels. This implied that annotation of the identified variants plays a major role in the analysis of mutational profiles. Literature implies that the amino acid substitutions that are deleterious as per the SIFT algorithm point towards the phenotype that is affected [33]. Thus, the results obtained through this analysis can be utilized for the identification of various plausible disease-causing genes. Furthermore, 407,952 non-coding variants were observed, along with 317,623 synonymous, 254,675 non-synonymous, 595 start lost, 2105 stop gain and 512 stop loss alterations (Fig. **4B**). Rare non-synonymous mutations in several genes are known to be accountable for colorectal tumors as per certain epidemiologic studies [34]. In the present study, most non-synonymous mutations were identified in SRR15987790 (38,093). Thus, further analysis of the non-synonymous variants identified can help understand the major genes that can predispose to colorectal cancer.

Likewise, to cross-verify and build the mutational profile further, summary files generated from PolyPhen2 were also analyzed. A total of 89,466 variants were benign, 10,005 were possibly damaging, and 13,177 were found to be probably damaging (Fig. **4C**). Moreover, 1901 possible damaging mutations and 2461 probably damaging alterations were noted in SRR15987795, most among all other exomes. Further comprehension into these might reveal the possible and probable genes that could be involved in colorectal tumors. This mutational profile sheds light on the types of mutations and exomes that must be looked at in much more depth and can help in unearthing rare or commonly overexpressed genes for better cancer prognosis. A more detailed analysis for each exome sample is provided in Supplementary file **S2**. SIFT annotations summary 1 and 2, PolyPhen2 annotations summary, SNPs and Indels called during GATK processing, SnpEff annotations summary.

### 3.4 Analysis of Filtered Mutations and Gene Expression Profiling

The major SNPs and their associated genes with a frequency greater than 10 across all the exomes analyzed included rs1041935 (*LGALS8*), rs16604022 (*PDE4DIP*), rs12338 (*CTSB*), rs1045051 (*RAD17*), rs2071307 (*ELN*), rs11543244 (*CPNE1*), rs1799971 (*OPRM1*), rs11526468 (*SEMA4D*), rs729593 (*MUC4*), rs1061308 (*PDE4DIP*), rs17855988 (*ELN*) and rs2229125 (*ADRA1A*). It was observed that these mutations, as per SIFT and PolyPhen2 analysis, were categorized into non-synonymous and deleterious variants. Furthermore, the variant rs1664022 in *PDE4DIP* was identified in all the exomes analyzed in this study. Table **2** shows the percentage frequency of occurrence of each of the shortlisted variants, their gene names and functions, gene symbols, UniProt IDs, dbSNP IDs and HGNC IDs.

#### 3.4.1 Gene Expression Profiling Using GEPIA

**Fig. (5):**
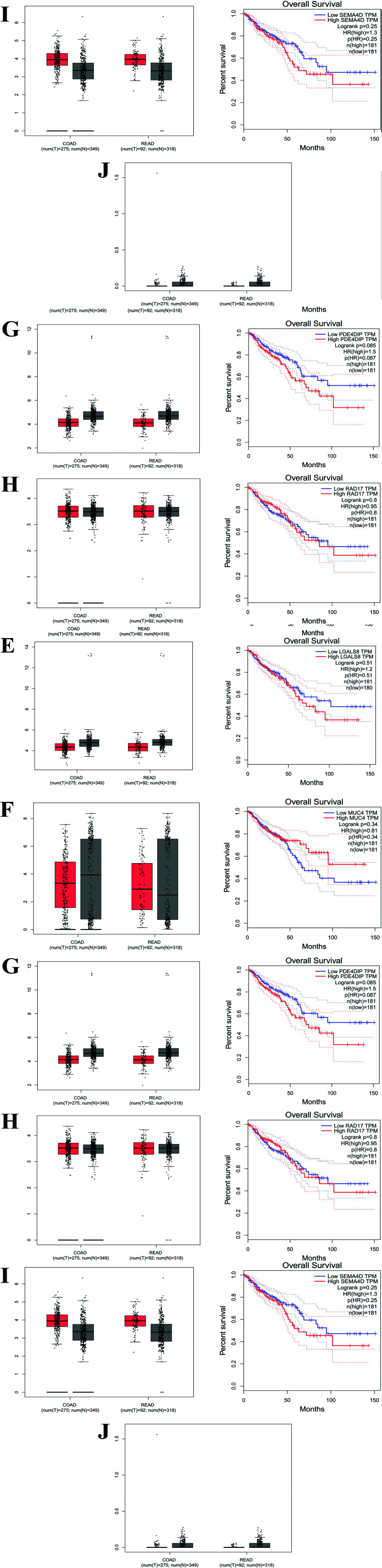
Box plots and predicted survival rates for the top ten shortlisted cancer genes with highly occurring mutations. The grey boxes represent the expression of the genes in normal tissues, and the red in tumor samples. The overall survival plots demonstrate that with high expression of specific genes, survival rates of patients begin to decrease. The survival rates are represented as months v/s percentage survival. **5A**) The expression and overall survival plot for *ADRA1*. **5B**) The expression and overall survival rate for *CPNE1*. **5C**) The expression and overall survival plot for *CTSB*. **5D**) The expression and survival rate for *ELN*. **5E**) The expression and overall survival study for *LGALS8*. **5F**) The expression and overall survival plot for *MUC4*. **5G**) The expression and overall survival rate for *PDE4DIP*. **5H**) The expression and overall survival plot for *RAD17*. **5I**) The expression and survival rate for *SEMA4D*. **5J**) The expression and overall survival study for *OPRM1*.

The box plots obtained for these top ten shortlisted cancer genes associated with their SNPs showed that out of 10 genes, 7 genes were found to have slightly more expression in tumor samples than in normal tissues, with grey boxes representing expression of the genes in normal tissues and red in tumor samples (Fig. **5**). Additionally, the overall survival plots demonstrated that with high expression of specific genes, the survival rates of patients begin to decrease (given in terms of months and percentage survival). Gene *ADRA1* was found to have a higher expression in normal tissues than in tumor samples. The overall survival plot also showed not much difference in survival when expressed in high or low quantities (Fig. **5A**). However, mutations in genes *CPNE1*, *CTSB* and *ELN* (Figs **5B**-**D**), indicated that their expression was slightly higher in tumor samples than in normal ones. Their survival plots also pointed out that higher expression of these genes may also reduce the percentage of survival, especially for mutations in gene *CTSB*. *LGALS8* was found to be expressed almost equally in both tumor and normal tissues (Fig. **5E**). Similar to *ADRA1*, gene *MUC4* was predicted to be expressed more in normal samples than in tumors, with survival predictions being less in patients with low rates of *MUC4* expression (Fig. **5F**). Genes *PDE4DIP*, *RAD17*, and *SEMA4D* had slightly more expression in tumors than normal tissues, with patient survival rates decreasing with increased expression of these genes with the mutations (Figs. **5G**-**I**). *OPRM1* was found not to be expressed in tumor samples but only slightly in normal ones (Fig. **5J**). No survival analysis was possible for this gene as expression was nil in tumor samples.

**Fig. (6):**
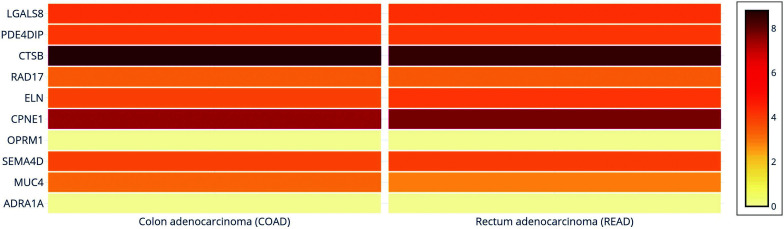
Differential gene expression of top ten identified genes against colon adenocarcinoma and rectum adenocarcinoma using GEPIA server. The mutated *CTSB* gene had the highest expression in both cancer types, followed by *CPNE1*. Genes *LGALS8*, *PDE4DIP*, *RAD17*, *ELN*, *MUC4* and *SEMA4D* had a mid-range expression in both cancer types, while *OPRM1* and *ADRA1* were the least expressed, thereby corroborating the results from our box plot and survival analysis. These results indicate that two major genes- *CTSB* and *CPNE1* may point towards causing colorectal cancers. Light brown indicates lower expression levels and dark brown to higher.

Since the expression levels were studied in two exomes- colon adenocarcinoma and rectum adenocarcinoma, a differential gene expression of these ten genes against the two cancer types showed that the mutated *CTSB* gene had the highest expression in both cancer types, followed by *CPNE1*. Genes *LGALS8*, *PDE4DIP*, *RAD17*, *ELN*, *MUC4* and *SEMA4D* had a mid-range expression in both cancer types, while *OPRM1* and *ADRA1* were the least expressed, thereby corroborating the results from our box plot and survival analysis (Fig. **6**). These results indicate that two major genes- *CTSB* and *CPNE1* may point towards causing colorectal cancers.

#### 3.4.2 Differential Gene Expression Against Various Cancers

**Fig. (7):**
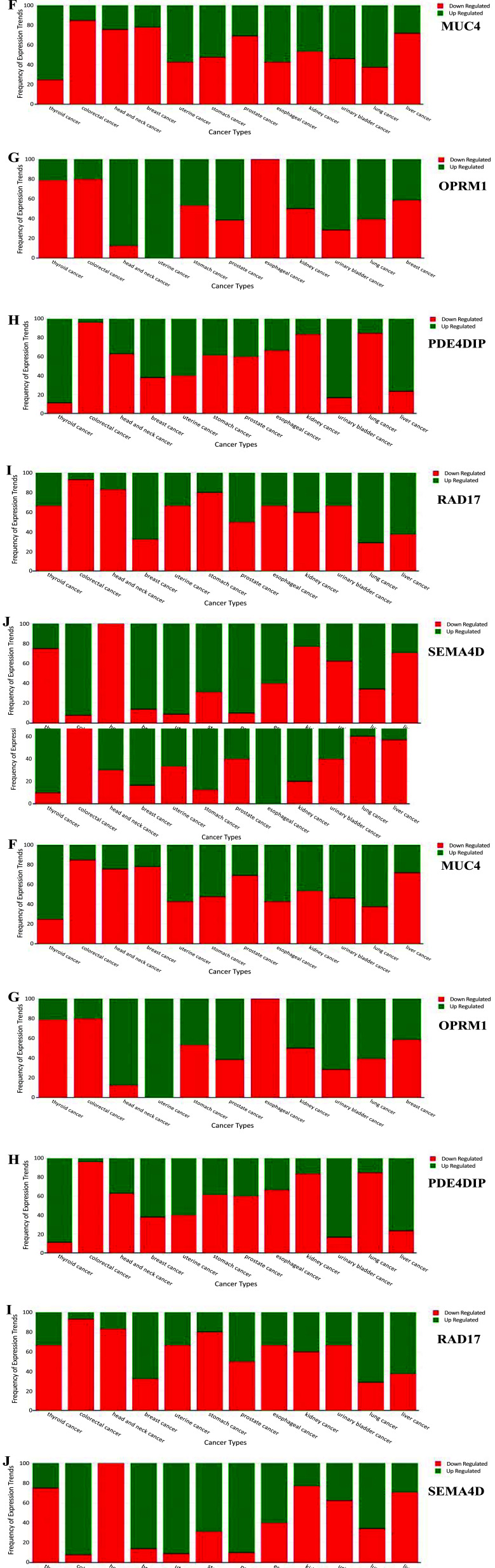
Differential gene expression of top ten genes with respect to various cancer types. This plot shows that the mutations identified in these genes may play a role in colorectal cancer and, if not, point towards their presence in implicating other cancer types, thereby allowing researchers and clinicians to focus more on these genes that predispose to specific cancer types. **7A**) Expression of *ADRA1*. **7B**) Expression of *CPNE1*. **7C**) Expression of *CTSB*. **7D**) Levels of *ELN* expression. **7E**) *LGALS8* expression levels. **7F**) Expression of*MUC4*. **7G**) Expression of *OPRM1*. **7H**) Expression of *PDE4DIP*. **7I**) Levels of *RAD17* expression. **7J**) *SEMA4D* expression levels.

To understand the possibility of these SNP-associated genes being over or under-expressed in not just colorectal cancer but in other types, a differential gene expression against various cancers revealed that *ADRA1* was completely down-regulated in colorectal cancer and in several other cancer types as well (Fig. **7A**), rendering this gene less likely to be involved in cancers. Corroborating the result we obtained from GEPIA gene expression studies, genes *CTSB* and *CPNE1* are highly overexpressed in colorectal cancer and most of the other cancer types in the OncoMX database, such as head and neck cancer, liver cancer, stomach cancer, kidney cancer, esophageal, lung and thyroid (Figs. **7B** and **C**). *ELN* was predicted to have overexpression in colorectal cancer but was under-expressed in the majority of the other types, while *LGALS8* was found to be downregulated in colorectal cancer and overexpressed in all other types (Figs. **7D** and **E**). Likewise, *MUC4* expression was down-regulated in all cancer types except for thyroid and lung cancer, indicating that it does not play a major role in causing colorectal cancer (Fig. **7F**). Surprisingly, *OPRM1* was downregulated in colorectal and most other cancers, but completely expressed in uterine cancers (Fig. **7G**). *PDE4DIP* and *RAD17* both do not play a major role in causing colorectal cancer, as observed and corroborated by gene expression profiling studies. However, it still may be upregulated in thyroid and uterine cancer (*PDE4DIP*) and lung cancer (*RAD17*) (Figs. **7H** and **I**). Contrary to the predictions obtained from GEPIA, *SEMA4D* was found to be upregulated highly in colorectal cancer and in several other types (Fig. **7J**). These results show that the mutations identified in these genes may play a role in colorectal cancer and, if not, point towards their presence in implicating other cancer types, thereby allowing researchers and clinicians to focus more on these genes that predispose to specific cancer types.

#### 3.4.3 Multi-dimensional Gene Analysis

When all 4535 colorectal cancer samples were run against our ten genes associated with mutations, plots of mRNA expression (RNA seq data) v/s the mutations in each gene were obtained. Fig. (**8**) illustrates in detail the different types of available and possible mutations in each gene against the available colorectal patient database. A few missense mutations were predicted for *ADRA1*, *CPNE1*, *CTSB*, *ELN*, *LGALS8*, *OPRM1*, *RAD17*, *PDE4DIP*, and *SEMA4D*. The maximum number of missense variations were noted in the *MUC4* gene, and most of these genes had shallow and deep deletions. There were very few amplifications in *CTSB*; however, a large number of deep and shallow deletions, as in *PDE4DIP*. *CPNE1* showed a very high number of amplifications. From this analysis, it is understood that two genes, *CTSB* and *CPNE1*, may tend to be more active in colorectal cancer cases than any other, implying further analysis into its use as a cancer indicator.

Additionally, a summary of all alterations per sample available in cBioPortal has been portrayed in Fig. (**9**), with different genetic alterations highlighted in various colors. All the samples were sorted by gene and type of the genetic event detected. Each query gene was represented as a row, and the samples as columns. The study of origin provided the list of all 13 colorectal cancer exomes. It was noted that in *LGALS8*, 1.1% of the samples that the gene was run against were altered, 5% of samples in *PDE4DIP*, and *CPNE1*, 3% in *CTSB*, *MUC4*, and *ADRA1*, 1.7% in *RAD17*, 1.5% in *ELN*, 1.2% in *OPRM1* and 1.9% in *SEMA4D*. These results corroborate the mRNA v/s mutation plots understood previously in Fig. (**8**), with most of the *PDE4DIP* mutations being missense, deep deletions in *CTSB* and amplifications in *CPNE1*. This multi-dimensional analysis provides a different perspective and validation of the major genes that are essential in colorectal cancer detection.

Further, Mutation data of each gene from cBioPortal is given as a different sheet in supplementary file **S3**. The study of origin, copy number, variant type, chromosome number, position of mutation, annotation, *etc*., are elucidated in detail. Supplementary file **S4** details the mutual exclusivity data obtained from cBioPortal on all 10 identified genes with the associated mutations, with its *p*-value, q-value, log2 odds ratio and tendency. The tendency of the genes to co-occur in the same samples (positive values) or occur in different samples (negative values) is also detailed.

### 3.5 Multi-variate Analysis Using Linear Regression

The least squares method showed that the R ^2^ values (the correlation between gene expression and gene frequency) were observed to be 0.8809 (for β1), 0.8462 (for β2) and 0.9394 (for β3). Although the R ^2^ values could have been better, it could be due to the smaller sample size since only 10 genes were shortlisted and studied for their expression and frequency. Additionally, the *p* -values for the D'Agostino- Pearson omnibus (K2) test and Shapiro-Wilk (W) test were found to be 0.6704 and 0.5883, respectively, where both passed the normality test with an alpha of 0.05.

Fig. (**10**) shows the correlation matrix for gene expression and frequency and the violin plot for the normality of residuals v/s the predicted *p* values. Table **3** highlights the statistical analysis results.

## 4 DISCUSSION

The present study provides a comprehensive computational perspective for obtaining important genes that predispose to colorectal cancer. The quality check results showed that the selected samples passed the tests in major criteria. Since all samples passed the adaptor content test, no adaptor trimming was needed. A recent study also examined the cancer exomes for breast cancer sequences and employed quality checks such as FastQC and MultiQC for quality assessment. Their results showed that 33 samples passed the tests out of 54 reads, with 21 falling into the ‘warning’ category [46]. The present study demonstrated slightly different results; however, the overall outcomes fell into the ‘pass’ category. Additionally, previously a study was also carried out for hepatocellular carcinoma cDNA end sequencing read, wherein adaptor clipping was carried out followed by alignment using Bowtie2 to map with the hg38 reference genome [47]. The Burrows-Wheeler Aligner (BWA) aligns all the short reads against the reference [12, 48]. Since Bowtie2 works rapidly and has better sensitivity and accuracy due to the presence of full-text minute index and dynamic programming algorithms, the present study employed this tool. Our study proved to be slightly better as none of the sequences required adaptor trimming. Moreover, the reference genome used was hg19 in the present study, and valid results were obtained from the previous works. However, the exome sequences selected were different in the present study, as was the cancer type, providing our study an edge over the previous works. Furthermore, SAMtools is considered a widely used software for the analysis of high-throughput sequenced data as it has a higher performance and enhanced ability for file indexing and sorting and writing the BAM files from SAM easily [12, 13]. Thus, our study employed this tool for SAM to BAM conversion.

A study by Xu *et al.*, 2020 [49], stated that germline genomic patterns were associated with the risk of cancers. This study employed the use of the GATK pipeline, with Haplotype caller for calling the variants for exome sequences. A comparison with Mutect2 also revealed that there were no differences in the outcome reproducibility. Additionally, another study employed low-input whole exome sequences and variants and INDELs were called using Haplotype caller from the GATK pipeline. The variants associated with cancers were then identified. In this study, both somatic and germline variants were called [26]. Another recent work employed Haplotype caller from GATK to call variants and select germline variants that were predisposed to colorectal cancer [50]. These works corroborate the work done in the present study. Moreover, our study identifies the germline mutations that also tend to have an effect on cancer risk. Studies have previously shown that germline mutations also affect tumor progression, thereby increasing their risk. Germline variations affect the expression of the genes and contribute vastly to disease progression [51].

Studies have stated that GATK has an F-score of 0.978, which is the harmonic mean of recall and precision, thereby making this toolkit a very reliable one for variant calling [16]. Moreover, GATK identifies all possible mutations and performs remarkably well in recognizing the true SNPs in the cancer exomes, making it the preferable choice over other somatic variant callers [17]. A recent study followed an NGS pipeline on the exonic and intronic sequences of colorectal cancer and identified in a single step, all different gene variations [16]. Different genes and variants are associated with them, such as POLE, POLD1, MSH3 and NTHL1. The clinical relevance of these different genes is yet to be understood, however, from among several identified mutations. The present study identified thousands of variants in terms of SNPs, indels, transitions, transversions, missense, nonsense and silent mutations. However, the exomes used were different in our study and exome sequence analysis was performed. Furthermore, the number of variants identified prior to annotation was large, which helped build a mutational profile.

A study involving the comparison of nine different *in silico* tools for post-processing of variants concluded that SIFT (Sorting intolerant from tolerant) stood as the most effective tool when taking into consideration the parameters of accuracy, sensitivity and specificity [52, 53]. SIFT classifies the variants into various classes such as “deleterious,” “tolerated,” “deleterious low confidence,” and “tolerated low confidence” [22, 33, 54]. This tool also predicts if the amino acid substitutions that have led to the formation of variants have an impact on the structure and function of a protein. SIFT performs the prediction of all the effects of possible amino acid substitutions at every position in the sequence [21]. The annotation of variants in the present study revealed tolerated, deleterious, non-coding, synonymous, non-synonymous, benign, possible and probably damaging mutations, from which the top ten genes with associated SNP changes were identified as non-synonymous and deleterious. Studies have shown that synonymous mutations are also responsible for acting as drivers in colorectal cancers [55, 56]. However, the present study focused primarily on understanding and identifying the non-synonymous mutations that could possibly be involved in colorectal cancer. A previous study detected somatic non-synonymous mutations associated with colorectal cancer in regions of liver metastases, 76.7% of the total somatic mutations detected, suggesting the importance of non-synonymous genes [57]. Another recent research noted a maximum number of non-synonymous mutations from WES-generated data of colorectal cancers, which were particular to metastatic tumors [58]. These studies point towards the importance of non-synonymous mutations that could be used as an indicator for colorectal cancer detection.

Due to their property of completely altering the amino acids, the non-synonymous variations are considered to be highly deleterious in nature, be it in germline or somatic variants. Additionally, non-synonymous SNPs can have adverse effects on proteins, such as altering the phenotype and genotype that may be the cause of a disease as dangerous as cancer [59, 60]. Moreover, the association of non-synonymous mutations with colorectal cancers has had some evidences in the past, with mutations being identified from exons [61]. A study has previously identified an E403K mutation in the *MCAK* gene, a non-synonymous variation to be linked with triggering colorectal cancer [62]. More recently, a study suggested that rare non-synonymous variations were associated with an increased risk of colorectal cancers, with several mutations identified in various genes [63]. These studies implicate non-synonymous variations as important colorectal cancer triggers and thus, more detailed studies are needed to confirm this. However, in the present multi-dimensional study, we identified ten highly occurring non-synonymous, deleterious mutations in genes associated with these mutations, with two genes highly expressed every stage of the downstream expression analysis, implicating their usefulness in predisposing to colorectal cancer.

Additionally, Fig. (**4**) primarily shows the relative gene expression data in normal and tumor samples, and the predicted survival plots. As pointed out, for ELN, CTSB and CPEN1, the expression was higher than in normal samples and high expression reduced the rate of survival. The same was found to be true for RAD17 gene. However, upon further differential gene expression comparative analysis, it was noted that although all genes except OPRM1 and ADRA1A showed expression in colon and rectal adenocarcinoma, their levels of expression were not as high as CTSB and CPNE1, which demonstrated the maximum expression. These results were to further confirm, shortlist and identify those that showed high expression predictions from all conducted studies so as to have more reliability and reproducibility of the outcomes, and to evidence their involvement in colorectal cancers. Therefore, further *in-vitro* studies are indispensable, yet these outcomes pave the way for taking CTSB and CPNE1 forward for prospective studies. Computational analysis from both differential expression and survival plots highlighted CTSB and CPNE1, and therefore, these were implicated in colon and rectal cancers.

Previously, Yasuda *et al*., 2016 identified the mutation rs1045051 (on *RAD17*) to have an effect on colorectal cancer in a Japanese cohort [64]. Furthermore, expression of *LGALS8* in lung, prostate and colorectal cancer is considered to have potential prognostic capabilities, particularly in advanced stages in patients with distant metastases [65]. *CTSB* has also been identified as a potential target for colorectal cancer therapy, owing to its ability to contribute to tumor development and invasion [66]. The overexpression of *CPNE1* in tumors has been implicated in promoting the progression of colorectal cancer and metastasis [67]. Similarly, *SEMA4D* has also been studied to understand its role in various human malignancies, including breast, colon and pancreatic cancer [68]. The missense variant, rs1664022, in *PDE4DIP* was identified in all the exomes analyzed in the present study, suggesting the possibility of utilizing it as a potential indicator for colorectal cancer. However, the role of *PDE4DIP* is poorly understood in the context of cancer, and further studies are required to validate the role of this particular SNP in *PDE4DIP* in colorectal cancer. With some of these results corroborating the outcomes of the present study, our study implicates *CTSB* and *CPNE1* as important colorectal cancer indicators and suggests further experimental validation and comprehensive analysis for prospective studies. Our aim was to focus on the identification of variants that could possibly point towards the risk of colorectal cancer. This was thus achieved with supporting results of the high expression of germline variants in *CTSB*, and *CPNE1* predicted in colorectal cancer. The present work points towards results that will also have to be tested further *in vitro* to conclude the predictions.

## CONCLUSION

Utilizing a comprehensive computational investigative approach to identify germline mutations in colorectal cancer exomes, this study revealed essential non-synonymous and deleterious SNPs that could potentially predispose to colorectal cancer. The present work utilized quality control tools such as FastQC and MultiQC,and filtered the variants generated using SIFT and PolyPhen2 that successfully categorized the mutations into synonymous, non-synonymous, start loss and stop gain mutations as well as marking them as possibly damaging, probably damaging and benign. Our work involved prioritizing the non-synonymous, deleterious SNPs since these polymorphisms bring about a functional alteration to the phenotype. The top variations associated with their genes with the highest frequency of occurrence included *LGALS8*, *CTSB*, *RAD17*, *CPNE1*, *OPRM1*, *SEMA4D*, *MUC4*, *PDE4DIP*, *ELN* and *ADRA1*A. An in-depth multi-dimensional downstream analysis of all these genes in terms of gene expression profiling and analysis and differential gene expression with regard to various cancer types revealed *CTSB* and *CPNE1* as highly expressed and overregulated genes in colorectal cancer. Several mutations were found to be predicted in these two genes in 4535 colorectal cancer *in-silico* patient analysis, which further validated our findings, warranting further experimental analysis as prospects for future studies. Our work sheds detailed light on the various alterations that might possibly lead to colorectal cancer and suggests further analysis into these genes to conclude the same.

## Figures and Tables

**Fig. (1):**
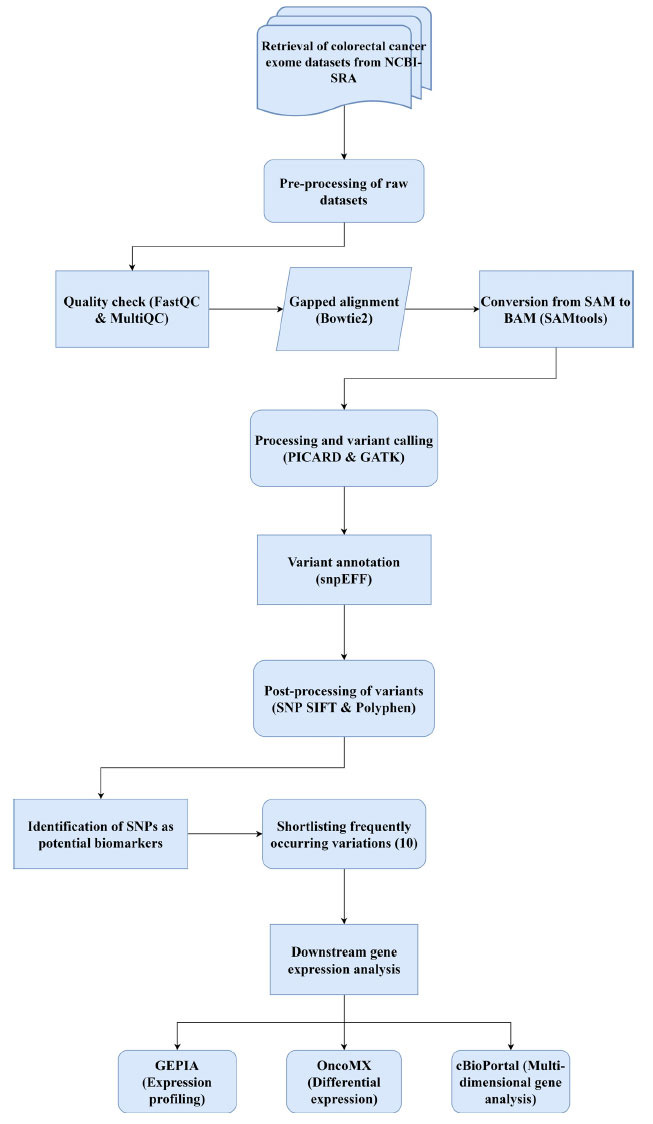
Flowchart illustrating the protocol used for the entire study. All tools used in the figure have been cited in the reference section.

**Fig. (8):**
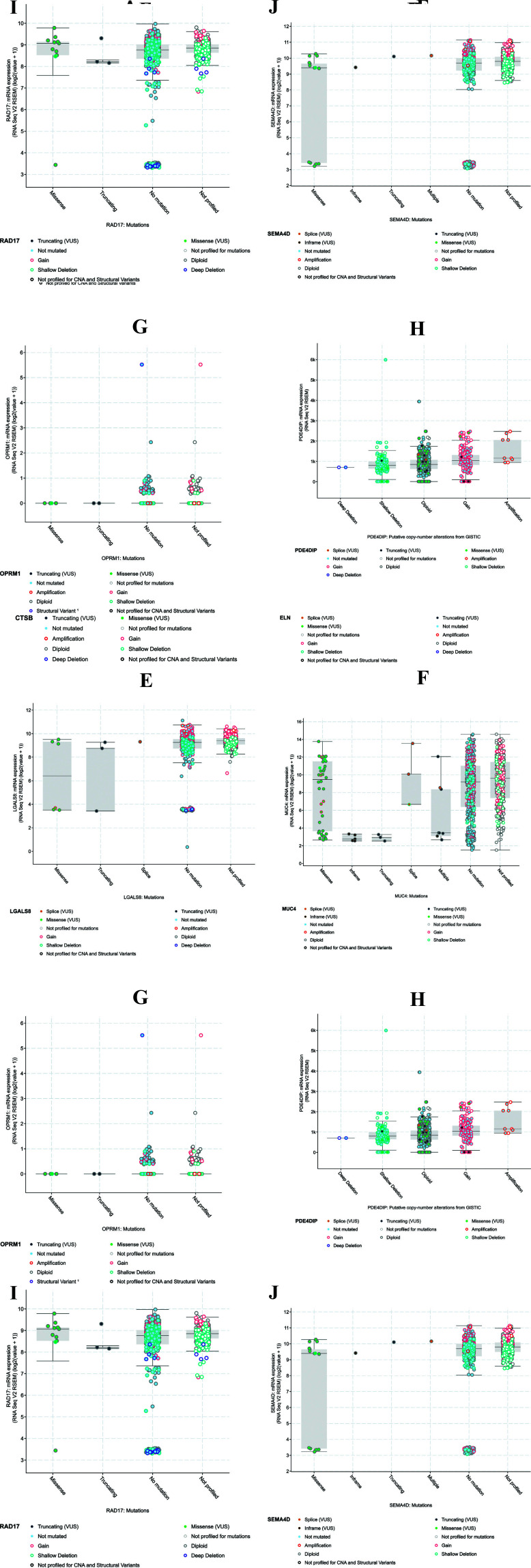
Different types of available and possible mutations in each gene against 13 available colorectal patient exomes in cBioPortal, with 4535 samples. Plots of mRNA expression (RNA seq data) v/s mutations in each gene revealed few missense mutations for *ADRA1*, *CPNE1*, *CTSB*, *ELN*, *LGALS8*, *OPRM1*, *RAD17*, *PDE4DIP*, and *SEMA4D*. The maximum number of missense variations were noted in the *MUC4* gene, and most of these genes had shallow and deep deletions. There were very few amplifications in *CTSB*. However, a large number of deep and shallow deletions, as in *PDE4DIP*. *CPNE1* showed a very high number of amplifications. Two genes, *CTSB* and *CPNE1*, tend to be more active in colorectal cancer cases than any other, implying further analysis into its use as a cancer indicator.

**Fig. (9):**
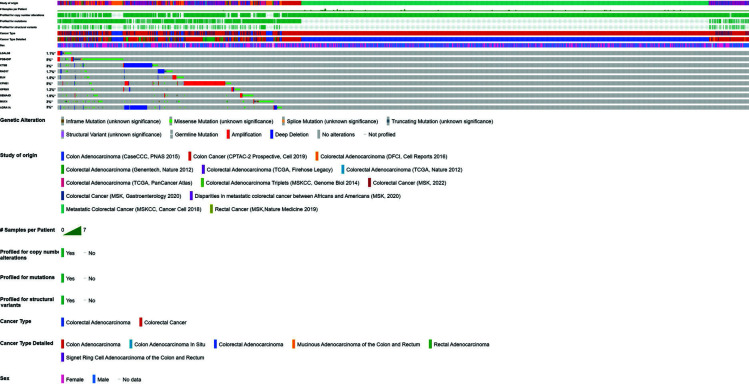
Summary of all alterations per sample available in cBioPortal. Different genetic alterations are highlighted in various colors. All the samples were sorted by gene and type of the genetic event detected. Each query gene was represented as a row, and the samples as columns. The study of origin provided the list of all 13 colorectal cancer exomes. It was noted that in *LGALS8*, 1.1% of the samples that the gene was run against were altered, 5% of samples in *PDE4DIP* and *CPNE1*, 3% in *CTSB*, *MUC4*, and *ADRA1*, 1.7% in *RAD17*, 1.5% in *ELN*, 1.2% in *OPRM1* and 1.9% in *SEMA4D*.

**Fig. (10):**
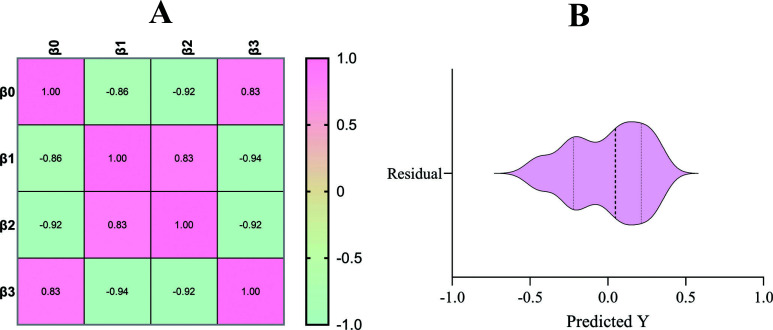
The correlation matrix for gene expression and frequency and violin plot for the normality of residuals v/s the predicted *p* values.

**Table 1:** Details of colorectal cancer exomes used in the study.

**Sl. No.**	**Colorectal Cancer Dataset**	**Design**	**Tissue**	**Isolate**	**Sex**	**Age**	**BioProject**	**BioSample**
1.	SRR14684620	xGen System	FFPE*	CC_9	Male	76	PRJNA733593	SAMN19416430
2.	SRR15987777	Illumina TruSeq Exome	FFPE	P9	NA	-	PRJNA764756	SAMN21527883
3.	SRR14463450	SureSelectXT reagent kit; all exon v5 probeset	-	Colon cancer cell	Male	62	PRJNA726023	SAMN18928492
4.	SRR15987799	Illumina TruSeq Exome	FFPE	P13	NA	-	PRJNA764756	SAMN21527863
5.	SRR15987796	Illumina TruSeq Exome	FFPE	P16	NA	-	PRJNA764756	SAMN21527866
6.	SRR15987795	Illumina TruSeq Exome	FFPE	P17	NA	-	PRJNA764756	SAMN21527867
7.	SRR15987790	Illumina TruSeq Exome	FFPE	P20	NA	-	PRJNA764756	SAMN21527871
8.	SRR15987786	Illumina TruSeq Exome	FFPE	P24	NA	-	PRJNA764756	SAMN21527875
9.	SRR15987785	Illumina TruSeq Exome	FFPE	P25	NA	-	PRJNA764756	SAMN21527876
10.	SRR15987792	Illumina TruSeq Exome	FFPE	P19	NA	-	PRJNA764756	SAMN21527869

**Abbreviation:** *FFPE: Formalin-Fixed Paraffin-Embedded

**Table 2:** Mutations and their associated genes and their functions, HGNC IDs, UniProt IDs, and dbSNP IDs with the highest frequency of occurrence across the ten colorectal cancer exomes analyzed.

**Gene Symbol**	**HGNC ID**	**Gene name**	**UniProt ID**	**Function**	**dbSNP ID**	**Frequency of Occurrence**
*LGALS8*	6569	Galectin 8/lectin, galactoside-binding, soluble, 8	O00214	A lectin with beta-galactoside-binding properties serves as a detector of membrane damage induced by infections. It hinders the growth of invading pathogens by directing them toward autophagy (Thurston *et al.*, 2012; Staring *et al.*, 2017) [35, 36].	rs1041935	80%
*PDE4DIP*	15580	Phosphodiesterase 4D interacting protein	Q5VU43	Serves as a tether, capturing elements of the cAMP-dependent pathway and localizing them to the Golgi and/or centrosomes (Mani *et al*., 2022) [37].	rs16604022, rs1061308	100%, 20%
*CTSB*	2527	Cathepsin B	P07858	A thiol protease is thought to play a role in the intracellular breakdown and renewal of proteins (Guo *et al.*, 2002) [38].	rs12388	60%
*RAD17*	9807	*RAD17* checkpoint clamp loader component/*RAD17* homolog (*S. pombe*)	O75943	Crucial for continual cell growth, preservation of chromosomal stability, and the activation of ATR-dependent checkpoints in response to DNA damage. Exhibits a modest ATPase activity necessary for chromatin binding. Plays a role in recruiting the RAD1-RAD9-HUS1 complex and RHNO1 to chromatin, and contributes to the activation of CHEK1. Additionally, it may function as a detector of DNA replication progression and be implicated in homologous recombination (Li *et al.*, 1999) [39].	rs1045051	40%
*ELN*	3327	Elastin	P15502	Primary structural protein is found in tissues like the aorta and nuchal ligament, where rapid expansion and complete recovery are essential. Functions as a molecular factor in the final stages of arterial morphogenesis, contributing to the stabilization of arterial structure by modulating the proliferation and organization of vascular smooth muscle (Keeley *et al.*, 2002) [40].	rs2071307, rs17855988	50%, 20%
*CPNE1*	2314	Copine 1	Q99829	A phospholipid-binding protein activated by calcium is involved in regulating intracellular processes mediated by calcium (Tomsig *et al.*, 2004) [41].	rs11543244	30%
*OPRM1*	8156	Opioid receptor mu 1	P35372	A receptor responsive to endogenous opioids like beta-endorphin and endomorphin (Pan *et al.*, 2003) [42].	rs1799971	50%
*SEMA4D*	10732	Semaphorin 4D	Q92854	A receptor located on the cell surface for PLXNB1 and PLXNB2 plays a crucial role in mediating cell-cell signaling (Janssen *et al.*, 2010) [43].	rs11526468	30%
*MUC4*	7514	Mucin 4, cell surface associated	Q99102	A mucin anchored to the membrane, belonging to a family of extensively glycosylated proteins that form the predominant constituent of mucus. Mucus is the slippery and thick secretion that covers epithelial surfaces (Moniaux *et al.*, 2000) [44].	rs729593	40%
*ADRA1A*	277	Adrenoceptor alpha 1A	P35348	This alpha-adrenergic receptor exerts its effects through interaction with G proteins, which in turn activate a phosphatidylinositol-calcium second messenger system. G(q) and G11 proteins mediate its effects. Nuclear ADRA1A-ADRA1B heterooligomers play a regulatory role in phenylephrine (PE)-stimulated ERK signaling in cardiac myocytes (Wright *et al.*, 2008) [45].	rs2229125	20%

**Table 3:** Multivariate analysis for correlation between gene expression data and gene frequency.

** Regression Type **	** Least Squares **		** - **
** Dependent Variable **	** Colorectal Cancer Label **		
**Regression type**	**Least squares**	**R ^ 2 ^ with other variables**	
β0	Intercept	-	-
β 1	Gene expression	0.8809	-
β 2	Gene frequency	0.8462	-
β 3	Gene expression: Gene frequency	0.9394	-
**Normality of Residuals**			
**Normality of Residuals**	**Statistics**	*** P * value**	**Passed normality test (alpha=0.05)**
D'Agostino-Pearson omnibus (K2)	0.7998	0.6704	Yes
Shapiro-Wilk (W)	0.9431	0.5883	Yes

## Data Availability

The authors confirm that the data supporting the findings of this research are available within the article.

## LIST OF ABBREVIATIONS

BWA: Burrows-Wheeler Aligner
BWT: Burrow-Wheeler Transformation
MSI: Microsatellite Instability
NCBI SRA: National Centre for Biotechnology Information, Sequence Read Archive
NGS: Next-generation Sequencing Technology
SIFT: Sorting Intolerant from Tolerant
VCF: Variant Calling Files
WES: Whole Exome Sequencing
